# Single and Multi-Objective Optimization of a Three-Dimensional Unbalanced Split-and-Recombine Micromixer

**DOI:** 10.3390/mi10100711

**Published:** 2019-10-21

**Authors:** Wasim Raza, Sang-Bum Ma, Kwang-Yong Kim

**Affiliations:** Department of Mechanical Engineering, Inha University, Incheon 22212, Korea; wasimkr@live.in (W.R.); msb927@inha.edu (S.-B.M.)

**Keywords:** micromixers, unbalanced split-and-recombination, surrogate modeling, Navier–Stokes equations, single and multi-objective optimizations, mixing index

## Abstract

The three-dimensional geometry of a micromixer with an asymmetrical split-and-recombine mechanism was optimized to enhance the fluid-mixing capability at a Reynolds number of 20. Single and multi-objective optimizations were carried out by using particle swarm optimization and a genetic algorithm on a modeled surrogate surface. Surrogate modeling was performed using the computational results for the mixing. Mixing and flow analyses were carried out by solving the convection–diffusion equation in combination with the three-dimensional continuity and momentum equations. The optimization was carried out with two design variables related to dimensionless geometric parameters. The mixing effectiveness was chosen as the objective function for the single-objective optimization, and the pressure drop and mixing index at the outlet were chosen for the multi-objective optimization. The sampling points in the design space were determined using a design of experiment technique called Latin hypercube sampling. The surrogates for the objective functions were developed using a Kriging model. The single-objective optimization resulted in 58.9% enhancement of the mixing effectiveness compared to the reference design. The multi-objective optimization provided Pareto-optimal solutions that showed a maximum increase of 48.5% in the mixing index and a maximum decrease of 55.0% in the pressure drop in comparison to the reference design.

## 1. Introduction

Microfluidics is rapidly emerging for precise control and manipulating fluids in microscale channels, and has found a wide range of applications in bioengineering, the chemical industry, and environmental monitoring [1,2,3]. Most applications of microfluidic systems involve the mixing of two or more species as a fundamental process. Successful completion of the applications requires rapid and efficient mixing, but microfluidic systems exhibit laminar flow characteristics due to their operation at low Reynolds numbers. The mixing achieved through molecular diffusion in the absence of turbulence is very slow and inefficient for microfluidic applications. Hence, it is crucial to attain efficient mixing to enhance the performance of microfluidic devices. 

Computational fluid dynamics (CFD) is extensively employed as a tool to evaluate the performance and the flow structure in micromixers. Over the past two decades, various passive micromixer designs [4,5] have been proposed based on mixing strategies such as parallel/sequential lamination [6,7], hydrodynamic focusing [8], and chaotic advection [9]. Combinations of these strategies can also be employed [10,11]. The sequential processes of splitting, rejoining, and rearranging the flow in a split-and-recombine (SAR) micromixer enlarge the interfacial surface between the mixing species exponentially and shorten the diffusion path, thereby enhancing the mixing. Chen et al. [12] proposed a planar cascaded SAR micromixer that achieved a mixing index of over 90% in a Reynolds number range of 34.6–150. Sharp bends formed by the channel edges and triangular baffles created corner vortices and Dean vortices that promoted chaotic advection and enhanced mixing. 

Chaotic advection produces transversal transport, which causes exponential growth of the interfacial area between the fluids, and decreases the striation thickness, which significantly improves the mixing. Chaotic advection can be generated through various channel features such as curvatures [13], grooves [9], and obstacles [14] on the channel walls and crossing channels [15]. Ansari and Kim [13] proposed a planar asymmetrical SAR micromixer with curved sub-channels. The unbalanced collision of the mixing species and Dean vortices in the curved channels generated chaotic advection. 

Li et al. [16] proposed a planar asymmetric SAR (P-ASAR) micromixer with staggered major sub-channels by modifying the micromixer reported by Ansari and Kim [13]. The micromixer attained a mixing index of 0.86 at *Re* = 80. Raza and Kim [17] proposed a micromixer based on unbalanced collision. Three-dimensional (3D) steps were introduced in the sub-channels, and the flow and mixing characteristics of the micromixer were numerically investigated in a range of Reynolds number, 0.1–120. The proposed micromixer had steps in the major and minor sub-channels with four mixing units. The micromixer showed a mixing index higher than 0.86 for Reynolds numbers larger than 20. The mixing was aided by chaotic advection produced through Dean vortices in the sub-channels, the collision of unequal mass flux due to asymmetrical splitting and rejoining, and the 3D flow field due to the steps. The mixing was enhanced in comparison to previous micromixers at intermediate Reynolds numbers.

As a result of developments in computational power, design optimization using CFD has become an efficient tool for the design of micromixers [18,19,20]. Cortes-Quiroz et al. [20] obtained an optimized staggered herringbone groove micromixer that satisfies several objectives using CFD, the design-of-experiment (DOE) method, surrogate modeling, and a multi-objective genetic algorithm (MOGA). Numerical simulations were performed to evaluate the values of the objective function at the design points created by a DOE technique called the Taguchi method. A Pareto-optimal curve representing reciprocity between the pressure drop and mixing index was obtained at *Re* = 1 and 10 by applying MOGA to the response functions developed by a surrogate model. 

Afzal and Kim [21] optimized the geometric and flow parameters of a convergent-divergent sinusoidal wall micromixer to maximize the mixing index. Different surrogate models were built using the input of the mixing index values at the sampling points generated by Latin hypercube sampling (LHS). The models used were the radial basis neural network (RBNN), response surface approximation (RSA), and Kriging (KRG) models. Sequential quadratic programming was used on the surrogate surface to locate the optimum design. Hossain et al. [19] optimized a 3D SAR micromixer with four design variables and two objective functions at *Re* = 15. RBNN was selected as a surrogate technique to replicate the functions representing the objectives of the mixing effectiveness and mixing index. Afzal and Kim [22] optimized a sinusoidally varying walled micromixer using the pressure drop coefficient and mixing index as the objective functions. The Pareto-optimal designs were compared in terms of mixing effectiveness to select an efficient design.

Numerical optimization methods using 3D CFD have been quite successful in enhancing the performance of various micromixers. Furthermore, the micromixers involving unbalanced collisions [13,16,17] showed a greater dependency of the mixing on the various geometrical parameters. Flow features such as the collision of unequal mass fluxes from the sub-channels, Dean vortices in the sub-channels, and chaotic advection due to 3D steps in the micromixers are affected by the changes in the geometrical parameters. 

However, hardly any numerical optimization has been performed on this type of micromixer to obtain optimum geometrical parameters for efficient mixing. Hence, in the present study, single- and multi-objective optimizations were explored to upgrade the capability to homogenize mixing species inside the micromixer proposed by Raza and Kim [17] at *Re* = 20. Mixing effectiveness was considered to be the target for single-objective optimization, while both the pressure drop across the microchannel and mixing index at the exit were optimized in the multi-objective optimization. Surrogate models were generated for different objectives using the KRG method, and Pareto-optimal solutions for multi-objective objective optimization were obtained using MOGA.

## 2. Problem Formulation and Analysis Methods

**Figure 1:**
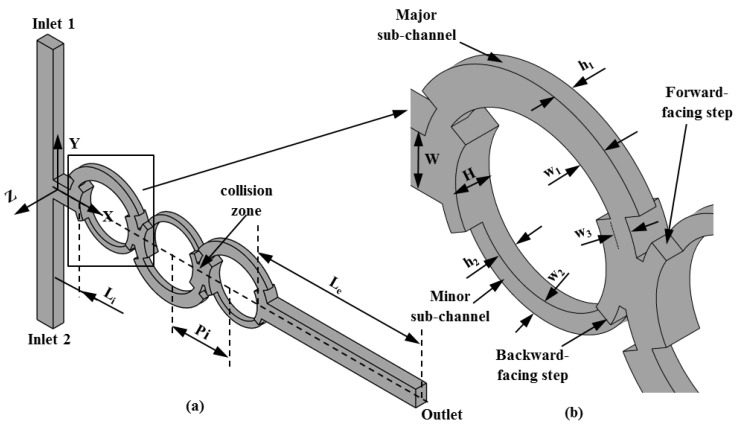
Diagrams of the micromixer configuration, reproduced with permission from [17]: (**a**) 3D view of the micromixer and (**b**) enlarged view of a mixing unit of the micromixer.

The micromixer configuration used for the optimization is shown in Figure 1. The micromixer consists of two side inlets connected to the main channel with three asymmetrical circular mixing units that undergo splitting-and-recombination. There are 3D steps in the sub-channels of the mixing units.

The dimensions of the reference design are the same as in previous work [17]: The axial length of the initial part of the main channel (*L_0_*), exit channel length (*L_e_*), total length (*L_t_*), pitch length (*Pi*), width of the main channel (*W*), width of the major sub-channel (*w_1_*), width of the minor sub-channel (*w_2_*), width of the dislocation (*w_3_*), depth of the major sub-channel (*h_1_*), depth of the minor sub-channel (*h_2_*), and total depth of the main channel (*H*) are 500 µm, 2950 µm, 6750 µm, 1200 µm, 300 µm, 200 µm,100 µm, 100 µm, 100 µm, 100 µm, and 200 µm, respectively. To analyze the mixing performance, the CFD code ANSYS CFX 15.0^®^ [23] was used for numerical analyses of the mass transport and fluid flow inside the micromixer model. The CFD code is based on finite-volume approximations and finds the solutions to the momentum and continuity equations for the Newtonian, incompressible, and steady 3D laminar flow:

(1) $$\nabla \cdot \vec{V}=0$$

(2) $$\left(\vec{V}\cdot \nabla \right)\vec{V}=-\frac{1}{\rho }\nabla P+\nu \nabla^{2}\vec{V}$$

where $\vec{V}\;$, *ν*, and *ρ* are the fluid velocity, kinematic viscosity, and density, respectively.

A dye-water solution and water enter inlets 1 and 2, respectively. Mass transport occurs in mixing due to diffusion and advection. Therefore, an advection-diffusion equation [24] was used to simulate the mass transport of fluids with constant properties during mixing:

(3) $$\left(\vec{V}\cdot \nabla \right)C=\alpha \nabla^{2}C$$

where *C* and *D* are the concentration and diffusivity coefficients, respectively. The diffusion coefficient was 1.2 × 10^−9^ m^2^/s. The properties of the two fluids are set to that of water at 25 °C (density *ρ* = 997 kg/m^3^ and viscosity *µ* = 0.00089 kg·m^−1^·s^−1^). Reynolds number was defined as *Re* = *ρUW*/*μ* where *U* is average velocity in the inlet channels.

The micromixer domain was discretized using a tetrahedral grid system. ANSYS ICEM CFD 15.0^®^ [23] was used to generate the mesh. Due to the unstructured tetrahedral elements, it is not possible to control the number of grid points in a specific direction. However, the average number of grid nodes used along *H* (Figure 1b) was 36. The boundary conditions were specified as follows: identical uniform velocities at the inlets, zero velocity at the walls, and zero static pressure at the outlet. For species transport, the molar concentration fractions of the dye-water solution were assigned a value of 1 at inlet 1 and 0 at inlet 2. Zero mass flux was employed at the channel walls.

The accuracy of the numerical results of mixing in the microchannel is sensitive to the discretization scheme. The discretization of the advection terms usually produces artificial diffusion due to truncation errors. The artificial diffusion cannot be fully ruled out but can be curtailed by employing certain methods [25]. Higher-order schemes such as the third-order QUICK [26] and second-order upwind schemes are less prone to numerical diffusion, in contrast to the first-order upwind scheme. Therefore, the second-order high-resolution scheme [23] was chosen for the discretization of the advection terms. A converged solution was obtained when the root-mean-square residual value of each variable was less than 1.0 × 10^−6^. The distribution of species at a plane perpendicular to the streamwise direction was used to measure the mixing capability. The variance of the mass fraction of species based on the concept of the intensity of segregation [27] was defined as follows:

(4) $$\sigma^{2}=\;\frac{1}{N}\overset{N}{\underset{i=1}{\sum }}{\left(c_{i}-{\bar{c}}_{m\;}\right)}^{2}$$

where *N, c_i_*, and ${\bar{c}}_{m}$ are the number of data points, the mass fraction at data point *i*, and the optimal mixing mass fraction, respectively. The mixing index at the plane was derived as follows:

(5) $$M=1-\sqrt{\frac{\sigma^{2}}{\sigma_{max}^{2}}}$$

where $\sigma_{max}$ is the maximum variance over the entire data range. A higher mixing index means a higher concentration uniformity. Therefore, for completely unmixed fluids, the mixing index is zero, while for completely mixed fluids, the value is unity. The mixing index at the exit (*M_o_*) was calculated on a plane located at 6700 µm from the main channel entry.

## 3. Design Variables and Objective Functions 

**Figure 2:**
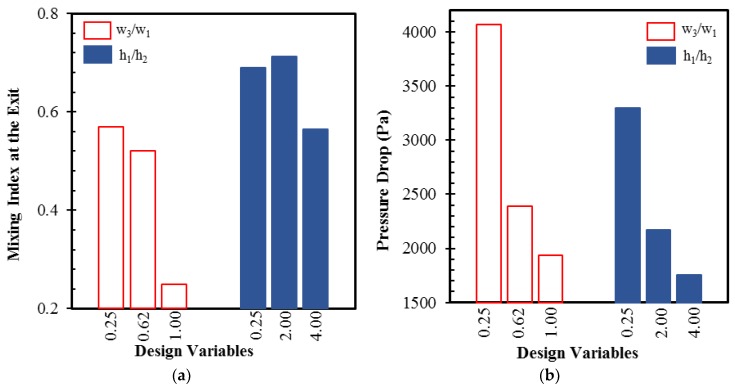
Responses of the objective functions to the design variables at *Re* = 20: (**a**) mixing index at the exit and (**b**) pressure drop.

The selection of appropriate design variables that largely affect the design objectives is an important step in the optimization process. The chosen design variables were the ratio of the width of the dislocation to the width of the major sub-channel (*w_3_*/*w_1_*) and the ratio of the depth of the major sub-channel to the depth of the minor sub-channel (*h_1_*/*h_2_*), as shown in Figure 1. The ranges for these variables were selected based on a preliminary parametric study. The parameters *W*, *H*, *w_1_*, and *w_2_* were kept constant throughout the optimization. Figure 2 shows the responses of the mixing and pressure drop to the changes of these design variables in the reference design of the micromixer at *Re* = 20. Variations of 128.5% and 26% in the mixing index are noticed with changes in *w_3_*/*w_1_* and *h_1_*/*h_2_*, respectively, within the tested ranges of the design variables. Similarly, there were variations of 110.2% and 88.5% in the pressure drop with changes in *w_3_*/*w_1_* and *h_1_*/*h_2_*, respectively. Table 1 lists the design variables and their ranges.

The objective functions for the optimization are the mixing index at the exit, mixing effectiveness, and pressure drop through the micromixer. The pressure drop is related to the pumping power necessary to drive the flow of mixing species and was determined as the difference between the area-averaged pressures at the inlet and exit of the main channel. The mixing effectiveness takes into account both the mixing index at the exit and the pressure drop in a single parameter as follows:

(6) $$ME=\frac{M_{0}}{K}$$

where the pressure loss coefficient (*K*) is calculated as follows:

(7) $$K=\frac{\Delta P}{0.5\rho U^{2}}$$

*U*, *ρ*, and *ΔP* are the average inlet velocity, density, and pressure drop, respectively.

The present work suggests two different approaches to deal with two objectives, i.e., enhancing mixing index and reducing pressure drop, in the optimization: one is to combine these two objectives into a single-objective function and to perform a single-objective optimization, and the other is to perform a multi-objective optimization with two objective functions related to these objectives. Therefore, two different optimizations were performed in this work: single-objective optimization for maximum mixing effectiveness and multi-objective optimization to find optimum solutions between the mixing index and the pressure drop.

The single-objective optimization uses an objective function *F_ME_* based on the mixing efficiency as follows:

(8) $$F_{ME}=-\left(ME\right)$$

The negative sign was introduced to define the optimization problem as a minimization of the objective function. The multi-objective optimization was done using objective functions *F_MI_* and *F_ΔP_*, which were defined using the mixing index at the exit (*M_0_*) and pressure drop (Δ*P*), respectively:

(9) $$F_{MI}=-\left(M_{0}\right)$$

(10) $$F_{\Delta P}=\left(\Delta P\right)$$

The values of different objective functions attained for the reference geometry are shown in Table 2.

## 4. Single and Multi-objective Optimization Methods

**Figure 3:**
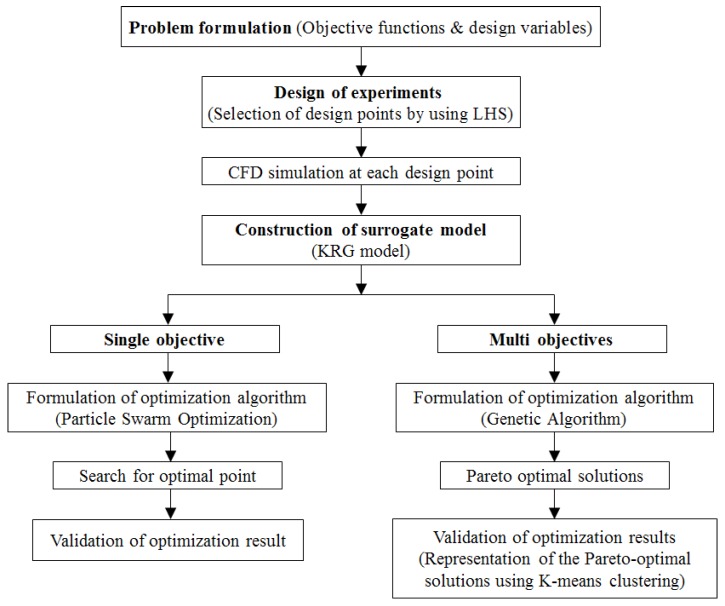
Single and multi-objective optimization procedures.

Figure 3 shows the optimization process used for the single and multi-objective optimizations based on surrogate modeling. The optimization problem was formulated as follows:

Objective functions: F(x) = [F1(x), F2(x), F3(x)…, Fn(x)]
Bounds of design variables: LB ≤ x ≤ UB, x ∈ R

where ***F(x)*** represents the objective functions, ***x*** is the vector of design variables, and ***LB*** and ***UB*** are the lower and upper bounds of the design variables, respectively [28]. 

DOE was used to select arbitrary points in the design space to construct surrogate models. DOE methods can be generally classified into two categories: orthogonal design and random design. The orthogonality of a design means that the model parameters are statistically independent. This indicates that the factors in an experiment are uncorrelated and can be varied independently.

A factorial design, represented by the orthogonality design, has some disadvantages: initially it is usually unclear which factor is important. Since the underlying function is deterministic, there is a possibility that some of the initial design points collapse and one or more of the time-consuming computer experiments become useless. This issue is called the collapse problem [29]. Most classic DOEs, including the factorial design, are only applicable to rectangular design regions. To overcome this problem, a random design is used. A random design means that the model parameter values for the experiments are assigned on the basis of a random process [30]. Thus, the collapse problem does not occur with the random design. This is because if one or more factors appear not to be important, every point in the design still provides some information regarding the influence of the other factors on the response. In this way, none of the time-consuming computer experiments will turn out to be useless [30].

Therefore, in the present work, LHS [31] as the random design was used to construct surrogate models which were created to approximate the objective functions. LHS is an effective sampling technique that uses an m × n simulation matrix where m is the number of levels (sampling points) to be examined and n is the number of design parameters. Each of the n columns of the matrix containing the levels, 1, 2, … , m, is randomly paired to form a Latin hypercube. This approach produces random sample points, ensuring that all portions of the design space are represented. A genetic algorithm (GA) [32] and Particle Swarm Optimization (PSO) [33] were used as searching algorithms to find the optimum points on the surrogate models in the design space. The MATLAB function *lhsdesign* was used to obtain the design points, and *maxmin* was used to maximize the minimum distance between adjacent design points [34]. LHS selected 12 design points for the two design variables, and the objective functions values at the design points were calculated numerically, as shown in Table 3.

The KRG model [35] was used for surrogate modeling of the objective functions. The KRG model is expressed by the unknown function ***y***(***x***) as follows:

(11) y(x) = f(x) + Z(x)

where ***x*** is an *m*-dimensional vector (for *m* design variables). ***f(x)*** is a known function representing the tendency for the design space and is generally expressed as a polynomial (also called a global model). ***Z(x)*** denotes the local deviations from the global model. In the KRG model, the local deviation at an unknown point can be expressed using a stochastic process. The sample points are interpolated with a Gaussian random function as a correlation function to estimate the trend of the stochastic process. The covariance matrix of ***Z(x)*** is given by:

(12) $$\mathrm{cov}\left[Z\left(x^{i}\right),\;Z\left(x^{j}\right)\right]=\sigma^{2}R\left[R\left(x^{i},\;x^{j}\right)\right],\;i,\;j\;=\;1,\;2,\ldots ,n_{s}$$

where ***R*** is a correlation matrix with a spatial correlation function (SCF) and $R\left(x^{i},\;x^{j}\right)$ as its elements. *σ*^2^ is the process variance, which is a scalar of the SCF that quantifies the correlation between any two n_s_ sampled data points *x*^i^ and *x*^j^ to control the smoothness of the KRG model. The Gaussian function is the most desirable SCF when used with gradient-based optimization algorithms because it provides a relatively smooth and infinitely differentiable surface [36].

A previous study [37] showed that PSO can find an optimal point with better performance in a single-objective optimization than GA. On the other hand, GA is suitable for relatively complex optimization problems such as multi-objective optimizations because it assesses various points in the design space. Therefore, PSO and GA were used for the single- and multi-objective optimizations, respectively. In PSO, the velocities and positions of the particles are constantly updated with the particles that previously had the best performance [33]. GA is a random global searching technique that finds an optimum for the fitness function by mimicking the evolutions observed in natural phenomena [32]. Based on growth and survival of the fittest, GA repeatedly searches for improved results.

MOGA was used to obtain Pareto-optimal solutions using the customized MATLAB function, “*gamultiobj”* [34]. The optimization used the GA to search the Pareto-optimal solutions in a feasible solution space bounded by the lower and upper bounds on the design variables. The function approximations for the two objectives, *F_MI_* and *F_∆p_*, were supplied in vector form. GA uses three main types of operation to create future generation from the current: selection, crossover and mutations [34]. In the single-objective optimization, the superiority of a solution over other solutions was easily determined by comparing their objective function values. However, in the multi-objective optimization problem, the goodness of a solution was determined by the dominance. Given the multi-objective optimization results, the non-dominated solution set of the entire feasible space is called the Pareto-optimal set and the boundary from the Pareto-optimal set is called the Pareto-optimal front. A solution is called non-dominated if none of the objective functions can be improved in value without degrading some of the other objective values. The function, *gamultiobj*, used in the present multi-objective optimization, was also coded to determine the dominance of solutions [38]. To identify among the Pareto-optimal solutions, these solutions were grouped into clusters using K-mean clustering [39].

## 5. Results and Discussion

**Figure 4:**
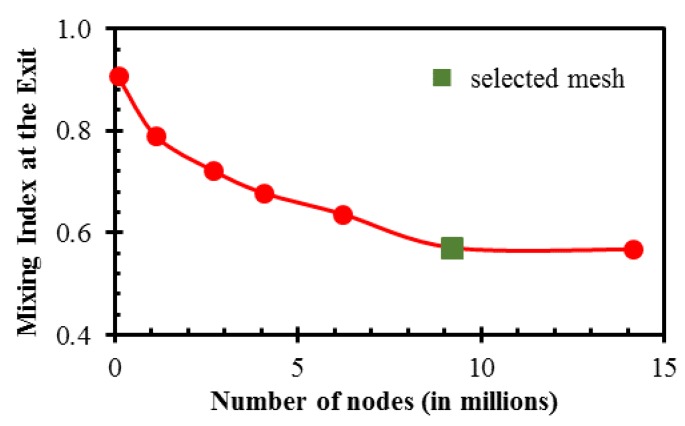
Mesh refinement test for mixing index at the exit at *Re* = 20.

A mesh test was performed to find the best grid system that ensures that the numerical results do not change upon further refinement. A mesh with 9.23 × 10^6^ nodes was chosen as an optimum mesh in a wide range of node numbers from 0.10 × 10^6^ to 1.41 × 10^7^, as shown in Figure 4. The mixing index at the microchannel exit for the selected mesh and a finer mesh show an insignificant difference of 0.52%.

Considering the methods outlined in the ref. [25], numerical diffusion was evaluated for the selected mesh. The numerical diffusion coefficient was 1.04 × 10^−8^ m^2^/s. Although this value is higher than the value of the molecular diffusion coefficient, the grid on further refinement shows a negligible change in the mixing index. This indicates that the mixing index changes negligibly with further reduction in the numerical diffusion, because the numerical diffusion coefficient is proportional to the mesh size. This may be caused by the dominance of chaotic advection over diffusion, and mixing is not attributed solely to diffusion. Furthermore, based on the numerical diffusion vs. mesh density graph [40], the required number of nodes to achieve the numerical diffusion coefficient equal or less than the molecular diffusion coefficient is approximately 3.41 × 10^8^ for this problem. Considering the computational limit, this is not possible at present.

**Figure 5:**
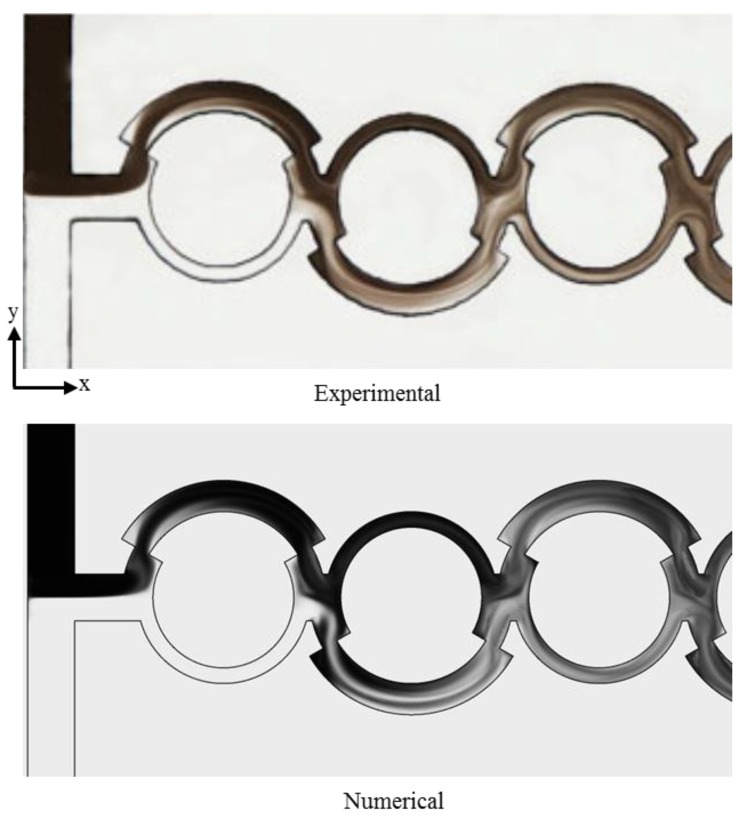
Validation of the numerical results in comparison with experimental data from Li et al. [16] for the dye mass fraction distribution at *Re* = 80.

The accuracy of the numerical simulation was verified by comparing the results with experimental data from Li et al. [16], as shown in Figure 5. The numerical plot of the dye mass fraction rendering of the micromixer was compared with the optical image of the dye mass fraction at *Re* = 80. There is acceptable agreement between the two concentration distributions, which validates the numerical approach.

Table 4 shows the results of the single-objective optimization for the mixing effectiveness. The numerical results at the optimal design indicate an improvement of 58.9% in the mixing effectiveness compared to the reference design. This indicates an enhancement of 3.7% in the mixing index at the exit and a reduction of 34.8% in the pressure drop in comparison to the reference design as a result of the optimization. The optimum design was obtained for *w_3_*/*w_1_* = 0.532 and *h_1_*/*h_2_* = 3.067, which are close to the middle range and upper bound, respectively. The surrogate model predicts the mixing effectiveness with a relative error of 2.5% in comparison to the numerical result with the optimal design.

**Figure 6:**
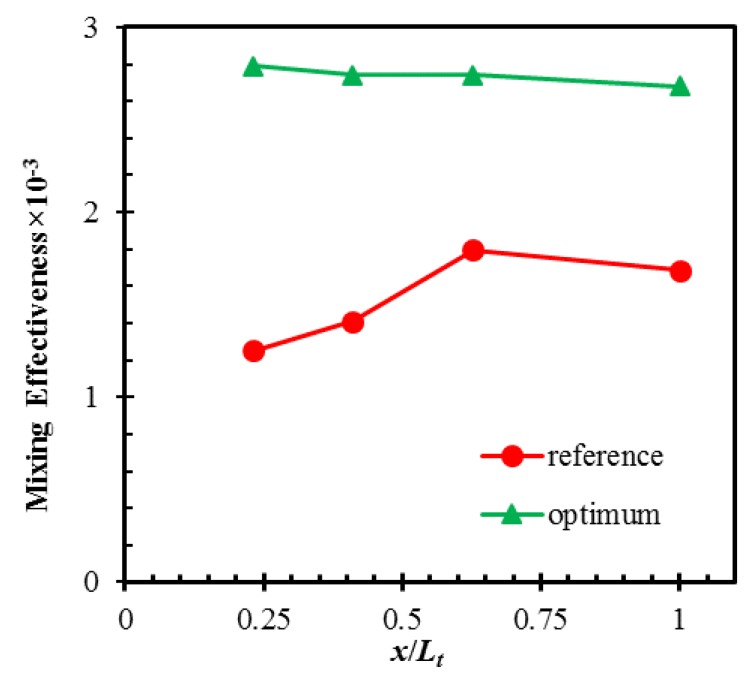
Developments of mixing effectiveness along the length of the reference and optimum micromixer designs (single-objective optimization).

Figure 6 shows a comparison of the mixing effectiveness in the axial direction for the reference and optimum micromixers. The mixing effectiveness was calculated on the y-z planes located after each mixing unit and at the exit (*x*/*L_t_* = 0.23, 0.41, 0.62, and 1). The mixing effectiveness is almost constant along the length of the optimized design, while it increases in the reference geometry. The optimum design shows much higher mixing effectiveness than the reference design over the entire length of the micromixer.

**Figure 7:**
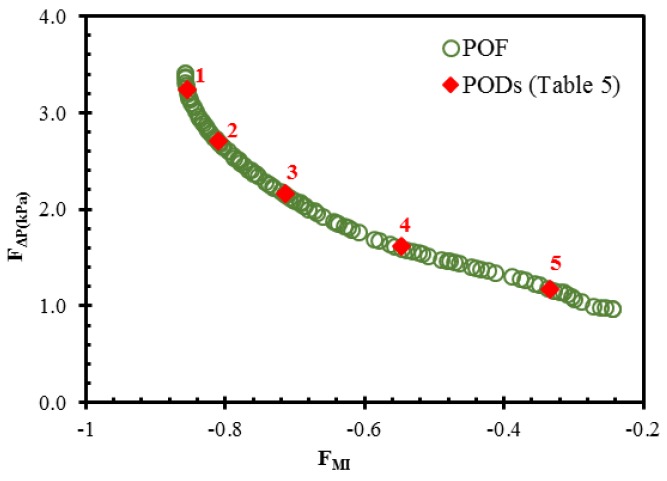
Pareto-optimal solutions (mixing index vs. pressure drop) obtained by multi-objective optimization.

Following the procedure in Figure 3, a multi-objective optimization was performed to find the Pareto-optimal solutions, as shown in Figure 7. The figure shows optimal compromises between the pair of conflicting objective functions: *F_MI_* and *F_ΔP_*. The concave feature of the Pareto-optimal front signifies that a gain in the mixing index occurs at the expense of a higher pressure drop. Each design corresponding to a solution on the Pareto-optimal front is a universally best design. Therefore, Pareto-optimal solutions provide a choice for the selection of the preferred combination of objective functions.

Five symbolic Pareto-optimal designs (PODs) were selected for analysis in terms of the two conflicting objective functions. These designs are marked on the Pareto-optimal curve in Figure 7 and listed in Table 5. POD 1 and POD 5 located at the extremes of the Pareto-optimal curve are mixing index-oriented and pressure drop-oriented designs, respectively. The fulfillment of one objective function causes deterioration in the competing objective function. The mixing index-oriented design is closer to the lower and upper limits of the design ranges for *w_3_*/*w_1_* and *h_1_*/*h_2_*, respectively.

**Figure 8:**
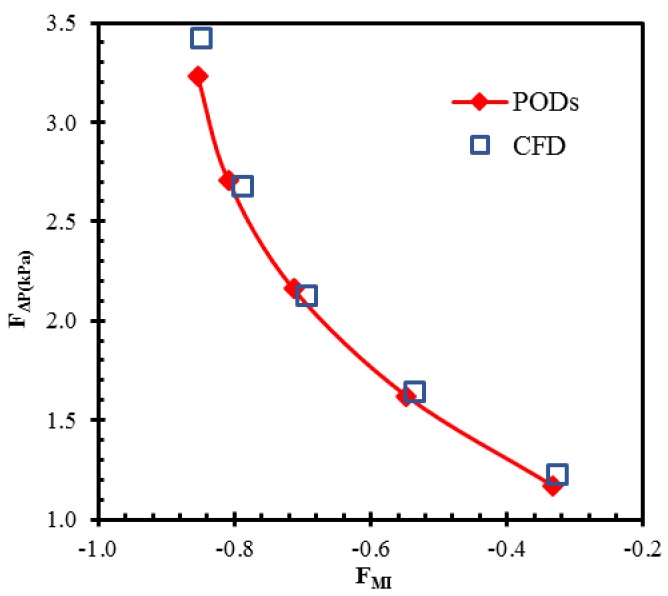
Comparison between MOGA predictions and numerical analysis results at selected PODs.

The objective function values predicted by the surrogate model are compared with the numerically calculated values for the representative PODs, as shown in Figure 8 and Table 5. The forecasted values of PODs show good agreement with the numerically calculated values for these PODs. The maximum relative errors in the surrogate prediction compared to the numerical results of the representative PODs were 2.7% and 5.9% for the mixing index at the exit and the pressure drop, respectively. The numerical result of POD 1 shows that a maximum improvement of 48.5% in the mixing index is achieved with POD 1 compared to the reference design. Similarly, a maximum reduction of 55.0% in pressure drop is achieved with POD 5 compared to the reference design. 

The literature studied indicates that single-objective and multi-objective optimizations have been carried on different micromixers but not compared. However, in this study, the comparison of results between single- and multi-objective optimizations of the micromixer indicates that the mixing index improvements achieved by the multi-objective optimization are higher than the single-objective optimization. Also, the pressure drop achieved by the multi-objective optimization is lower than the single-objective optimization result. Hence, the multi-objective optimization is more suited to the optimization of the micromixer performance.

**Figure 9:**
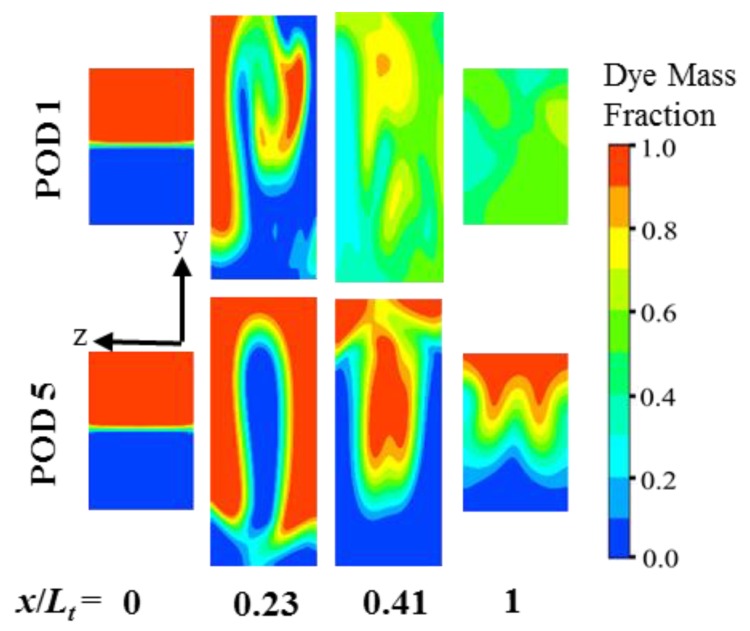
Dye concentration distributions on y-z planes for POD 1 and POD 5.

The dye concentration distributions are plotted on transverse planes along the length of the micromixer (*x*/*L_t_* = 0, 0.23, 0.41, and 1) for a qualitative comparison of the mixing between POD 1 and POD 5, as shown in Figure 9. Greater distortion of the fluid interfaces is observed for POD 1 due to the enhanced stretching and folding process, which extends the interfacial contact area for diffusion and thereby establishes a more uniform concentration distribution at a much shorter axial length than POD 5.

**Figure 10:**
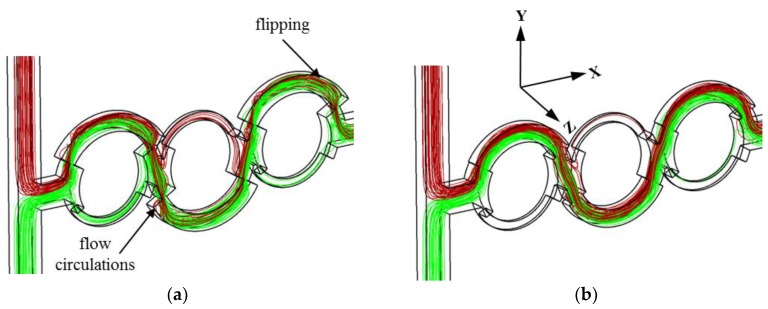
3D streamlines originating from the two inlets: (**a**) POD 1 and (**b**) POD 5.

The flow structure responsible for the dissimilar mixing qualities in POD 1 and POD 5 were investigated using the 3D streamlines of the fluids entering from the two inlets shown in Figure 10. The fluids from the two inlets come into contact at the T-joint. Distinct disparity in the streamlines is observed at the splitting of the flow. At the second joint, the flow from a single inlet splits into the major and minor sub-channels of the second mixing unit in POD 1, but most of the flow from the first major sub-channel passes through the second major sub-channel only in POD 5. The splitting of the flow into the two sub-channels in POD 1 causes increased stretching and folding of the fluid interface in the collision zone, as shown in Figure 10. The flipping of streamlines and flow circulation are not presented in POD 5, but are observed in the major sub-channels of POD 1, which accelerates the mixing. In addition, the smaller flow area at the dislocations of POD 1 than in POD 5 increases the local flow velocity, which also enhances the flow perturbation and thereby aids in mixing.

**Figure 11:**
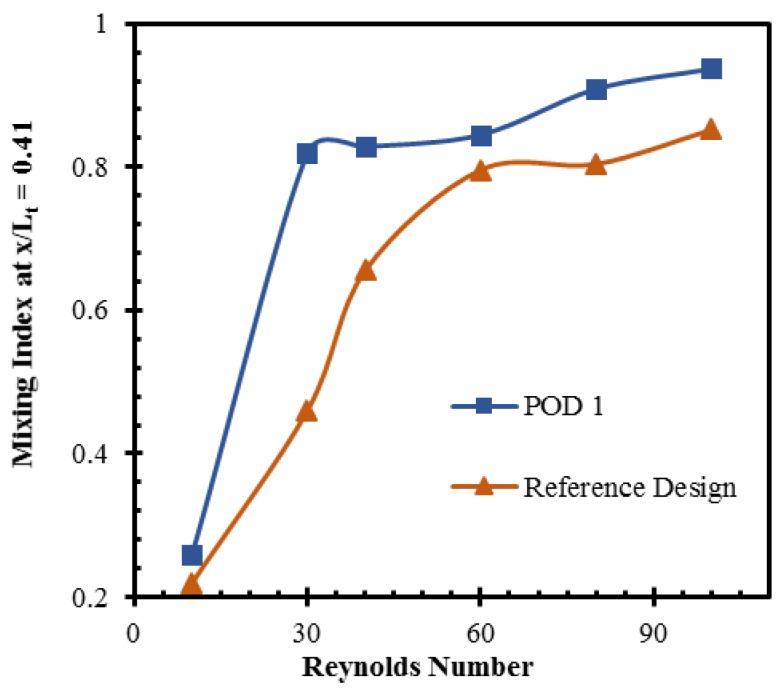
Comparison of mixing index at different Reynolds numbers between POD 1 and reference design.

The mixing capability of POD 1 is compared with that of the reference design at different Reynolds numbers, as shown in Figure 11. Both the reference micromixer and POD 1 achieve a mixing index over 0.90 at the exit for higher Reynolds numbers (*Re* = 60, 80, and 100). However, POD 1 achieves this mixing index at a much shorter length. Therefore, the mixing index at the end of the second mixing unit (*x*/*Lt* = 0.41) was chosen for comparison instead of the exit to clarify the improvement in the mixing. POD 1 shows better mixing capability than the reference design at all Reynolds numbers. This design shows improvements of 77.8% and 26.2% in the mixing index at Reynolds numbers of 30 and 40, respectively.

The mixing effectiveness (*ME*) [41], mixing cost (*MC*) [42], and mixing energy cost (*MEC*) [43,44] have been used as performance parameters in the literature to evaluate micromixers. Higher mixing effectiveness and MC and a lower value MEC represent better micromixer performance. The expressions for the MC and MEC can be written as follows:

(13) $$mixing\;cost=\frac{\eta }{\Delta P}$$

(14) $$mec=\frac{{\bar{C}}_{p}}{\eta }$$

where Δ*P*, ${\bar{C}}_{p},$ and *η* denote pressure drop, mean input power coefficient, and mixing efficiency, respectively. Mixing efficiency and mean input power coefficient (${\bar{C}}_{p}$) are written as follows:

(15) $$\eta =\left(1-\frac{\sigma }{\sigma_{max}}\right)\times 100$$

(16) $${\bar{C}}_{p}=2\Delta Pq/\rho V^{2}$$

where *σ*, $\sigma_{max}$, *ρ*, Δ*P*, *q*, and *V* are the standard deviation of the concentration, maximum standard deviation, density of fluid, pressure drop, dimensionless flow rate, and average flow velocity, respectively. The performance parameters of the representative PODs were compared with those of three previous micromixers involving unbalanced collisions, as shown in Table 6. All the PODs outperform the previous micromixers in terms of the mixing index at the exit, but POD 1 shows *ME* and *MC* values that are less than or equal to all micromixers except that proposed by Xia et al. [45] due to higher pressure drop. In the case of *MEC*, all the PODs except POD 1 and POD 5 show lower values than those of the previous micromixers. POD 4 shows the best values for *ME*, *MC*, and *MEC* among all the micromixer designs. This micromixer design shows 315%, 37%, and 38% higher values of the mixing index, *ME*, and *MC* than the lowest values shown by the other micromixer designs, respectively. 

## 6. Conclusions

Single and multi-objective optimizations of a 3D-ASAR micromixer were performed at Reynolds number of 20, using surrogate modeling in conjunction with mixing and flow analyses. The optimizations were carried out with two dimensionless design variables, *w_3_*/*w_1_* and *h_1_*/*h_2_*. The mixing effectiveness was selected as a single-objective function, while both the mixing index at the exit and pressure drop across the micromixer were chosen for the two-objective optimization. The KRG model was selected to generate the surrogate of the objective functions. 

The parametric study demonstrated that the mixing index at the outlet shows non-monotonic behavior and has maxima, but the pressure drop shows a monotonically decreasing behavior for the two design variables. An improvement of 58.9% in the mixing effectiveness was obtained in comparison to the reference design by the single-objective optimization. This enhancement corresponds to a 3.7% rise in the mixing index at the exit and a 34.8% decrease in the pressure drop in comparison to the reference design. The surrogate prediction error for the single-objective optimization was estimated to be 2.5%. 

The reciprocity between the two objective functions represented by the Pareto-optimal front for the multi-objective optimization was obtained by employing surrogate modeling and MOGA. The numerical analysis of five representative PODs indicated a maximum improvement of 48.5% in the mixing index for a mixing index-oriented design (POD 1) and a maximum reduction of 55.0% in the pressure loss for a pressure drop-oriented design (POD 5) in comparison to the reference design. On the other hand, POD 4 showed the best values for *ME*, *MC*, and *MEC* among all the micromixer designs, including three previous micromixers involving unbalanced collisions. The surrogate model predicted the mixing index and pressure drop with maximum relative errors of 2.7% and 5.9%, respectively. There were low percentages of improvement in the mixing index and the reduction in the pressure drop by the single-objective optimization in comparison to the multi-objective optimization, which indicates that the multi-objective optimization is much more favorable for improvement of the micromixer performance. 

## Figures and Tables

**Table 1:** Ranges of design variables.

Design Variable	Lower Bound	Upper Bound
*w_3_/w_1_*	0.25	1.00
*h_1_/h_2_*	0.25	4.00

**Table 2:** Reference design and its objective function values.

Reference Design				
Design Variables		Objective Functions		
*w_3_/w_1_*	*h_1_/h_2_*	Mixing Effectiveness, *ME*	Mixing Index at Exit, *M_o_*	Pressure Drop, Δ*P* (kPa)
0.50	1.00	1.687 × 10^−3^	0.571	2.719

**Table 3:** Design variables and objective function values at LHS design points.

Design Point	Design Variables		Objective Functions		
	*w_3_/w_1_*	*h_1_/h_2_*	Mixing Index, *M_o_*	Pressure Drop, ∆*P* (kPa)	Mixing Effectiveness, *ME* (×10^−3^)
1	1.000	3.318	0.265	1.111	1.92
2	0.386	3.659	0.731	2.423	2.42
3	0.318	2.295	0.837	3.368	1.99
4	0.455	1.273	0.699	2.702	2.08
5	0.727	1.614	0.419	1.756	1.92
6	0.591	0.250	0.690	3.276	1.69
7	0.659	4.000	0.399	1.344	2.39
8	0.523	2.636	0.640	1.892	2.72
9	0.795	2.977	0.328	1.289	2.04
10	0.932	1.955	0.302	1.404	1.73
11	0.864	0.591	0.291	2.652	0.88
12	0.250	0.932	0.590	4.028	1.18

**Table 4:** Single-objective optimization for maximum mixing effectiveness (*ME*).

Design Variables		Surrogate Prediction	Numerical Analysis			% Error
*w_3_/w_1_*	*h_1_/h_2_*	*ME*	*M_o_*	Δ*P* (kPa)	*ME*	*ME*
0.532	3.067	2.750 × 10^−3^	0.592	1.772	2.681 × 10^−3^	2.50

**Table 5:** Results of multi-objective optimization for selected Pareto-optimal designs (PODs).

Selected POD	Design Variables		Surrogate Prediction		Numerical Analysis	
	*w_3_/w_1_*	*h_1_/h_2_*	*M_o_*	Δ*P* (kPa)	*M_o_*	Δ*P* (kPa)
1	0.307	2.797	0.854	3.234	0.848	3.425
2	0.373	2.914	0.809	2.709	0.788	2.674
3	0.450	3.046	0.713	2.159	0.694	2.124
4	0.572	3.137	0.548	1.620	0.536	1.640
5	0.778	3.756	0.334	1.169	0.326	1.224
reference design	0.500	1.000	-	-	0.571	2.719

**Table 6:** Comparison of performance parameters among symbolic PODs and previous micromixers based on unbalanced collisions.

Parameters	PODs					Ansari and Kim * [13]	Xia et al. * [45]	Li et al. * [16]
	1	2	3	4	5			
*Mixing index*	0.848	0.788	0.694	0.536	0.326	0.178	0.129	0.229
Δ*P* (Pa)	3425	2674	2125	1640	1224	658	538	907
*ME* (×10^−3^)	1.99	2.37	2.62	2.63	2.14	2.17	1.92	2.03
*MC* (×10^−3^)	0.25	0.29	0.32	0.33	0.27	0.27	0.24	0.25
*MEC*	3.35	2.82	2.54	2.53	3.11	3.07	3.47	3.29

* calculated values for three mixing units.

## Nomenclature

*c*	mass fraction
*D*	diffusion coefficient (m^2^/s)
FBFS	forward- and backward-facing steps
*h_1_*	depth of major sub-channel (µm)
*h_2_*	depth of minor sub-channel (µm)
H	total depth of main channel (µm)
KRG	Kriging model
LHS	Latin hypercube sampling
*L_i_*	length of initial part of main channel (µm)
*L_e_*	exit channel length (µm)
*L_t_*	total length of the micromixer (µm)
*M*	mixing index
*M_0_*	mixing index at the exit
*MC*	mixing cost
*ME*	mixing effectiveness
*MEC*	mixing energy cost
MOGA	multi-objective genetic algorithm
*N*	number of sampling points
*P*	pressure drop (Pa)
*Pi*	pitch length (µm)
POD	Pareto-optimal design
POF	Pareto-optimal front
PSO	particle swarm optimization
*Re*	Reynolds number
*SAR*	split and recombine
*U*	average inlet velocity (m/s)
*V*	average velocity in main channel (m/s)
*W*	width of main channel (µm)
*w_1_*	width of major sub-channel (µm)
*w_2_*	width of minor sub-channel (µm)
*w_3_*	width of dislocation (µm)
x, y, z	Cartesian coordinates
**Greek Symbols**	
*α*	fluid diffusivity coefficient (m^2^/s)
*µ*	fluid dynamic viscosity (kg·m^−1^·s^−1^)
*ν*	fluid Kinematic viscosity (m^2^/s)
*ρ*	fluid density (kg/m^3^)
*σ*	standard deviation
**Subscripts**	
*i*	sampling point or fluid component
*m*	optimal mixing
*max*	maximum value
*x*	axial distance
